# Prediction of Depression in Cancer Patients With Different Classification Criteria, Linear Discriminant Analysis versus Logistic Regression

**DOI:** 10.5539/gjhs.v8n7p41

**Published:** 2015-11-03

**Authors:** Zahra Shayan, Naser Mohammad Gholi Mezerji, Leila Shayan, Parisa Naseri

**Affiliations:** 1 Department of Community Medicine, The Medical School, Shiraz University of Medical Sciences, Shiraz, Islamic Republic of Iran; 2 The Medical School, Saveh University of Medical Sciences, Saveh, Islamic Republic of Iran; 3 Trauma Research Center, Shiraz University of Medical Sciences, Shiraz, Islamic Republic of Iran; 4 The Medical School, Arak University of Medical Sciences, Arak, Islamic Republic of Iran

**Keywords:** comparison, linear discriminant analysis, logistic regression, prediction, classification criteria

## Abstract

**Background:**

Logistic regression (LR) and linear discriminant analysis (LDA) are two popular statistical models for prediction of group membership. Although they are very similar, the LDA makes more assumptions about the data. When categorical and continuous variables used simultaneously, the optimal choice between the two models is questionable. In most studies, classification error (CE) is used to discriminate between subjects in several groups, but this index is not suitable to predict the accuracy of the outcome. The present study compared LR and LDA models using classification indices.

**Methods:**

This cross-sectional study selected 243 cancer patients. Sample sets of different sizes (n = 50, 100, 150, 200, 220) were randomly selected and the CE, B, and Q classification indices were calculated by the LR and LDA models.

**Results:**

CE revealed the a lack of superiority for one model over the other, but the results showed that LR performed better than LDA for the B and Q indices in all situations. No significant effect for sample size on CE was noted for selection of an optimal model. Assessment of the accuracy of prediction of real data indicated that the B and Q indices are appropriate for selection of an optimal model.

**Conclusion:**

The results of this study showed that LR performs better in some cases and LDA in others when based on CE. The CE index is not appropriate for classification, although the B and Q indices performed better and offered more efficient criteria for comparison and discrimination between groups.

## 1. Introduction

Classification method in medical studies is important when researchers are interested in classifying subjects in specific groups according to specific characteristics. Multivariate analysis is commonly used to classify this type of data. Logistic regression (LR) and linear discriminant analysis (LDA) are two forms of multivariate analysis used to predict membership in two or more mutually exclusive groups using a set of predictors (Alkarkhi & Easa, 2008; Montgomery, White, & Martin, 1987).

LDA is similar to LR and both can be used in one study; nevertheless, the two methods differ in statistical assumptions. The underlying assumptions of LDA are the normal distribution of independent variables and equal variance-covariance matrices within each group. When some or all variables are categorical, the assumptions are nearly always violated. This is particularly important when the objective is to estimate the magnitude of the effects of the predictor variables. When the objective is only prediction or classification, these assumptions are less constraining and both methods provide the same model. LR does not have as many assumptions (Johnson & Wichern, 2007; Montgomery et al., 1987). When the assumptions of LDA are met, this type of analysis is more powerful than LR. LR is the common choice when all aspects are considered because the assumptions of LDA can rarely be met.

Several studies have compared the two models. One study indicated that LDA is more useful for classification of cases into several groups, while LR is more useful for relating a binary dependent variable to independent variables (Press & Wilson, 1978). Other studies have reported that LDA is asymptomatically more efficient than LR when the assumptions of multivariate normality and equal covariance hold (Barön, 1991; Efron, 1975).

Most studies on LDA have focused only on continuous variables (Barön, 1991; Lei & Koehly, 2003); however, categorical variables could also be useful predictors. There is little theory available to deal with this situation (Krzanowski, 1982, 1986; Lee, Song, & Lu, 2007). It has been recommended for the numerical value of a variable to equal one if the object possesses the characteristic and zero if the object does not. This variable is then treated like a continuous variable in the usual models (Johnson et al., 2007).

Classification error (CE; percentage of incorrectly classified observation) is a simple and common criterion used to compare two models; however, it is not a sensitive and statistically appropriate measure (Harrell, 1997). CE can be similar in two models, but can be overestimated when there is a difference between models. If the predicted value for one case is 0.51 and for another is 0.99, both will be classified into the same group, which demonstrates that CE does not reveal differences between values well. CE also cannot determine the accuracy of the predictions (Pohar, Blas, & Turk, 2004); thus, other methods should be employed for comparison two models. Four measures have been proposed to compare predictive accuracy of two methods (Harrell & Lee, 1985). Only Pohar et al. have investigated this subject thus far. They studied measures of predictive accuracy by simulation (2004). More studies are necessary to explore the difference between classification criteria.

The present study compared LR and LDA for classification of subjects to groups having different conditions using continuous and categorical variables and different indices to increase accuracy of the prediction based on sample size. Specifically, real data used to predict depression in cancer patients undergoing chemotherapy and radiotherapy.

## 2. Methods

The 243 subjects selected were patients in the chemotherapy and radiotherapy wards of Shiraz Nemazee Hospital. All subjects who agreed to participate in this research were selected. The patients had an incomplete data were excluded. The data was collected using two forms, one for demographic characteristics and one for medical conditions.

The independent variables were sex, marital status, education, location, income, satisfaction from her/his condition, family history of depression, type of cancer, knowledge about the disease, type of treatment (categorical variables), age, and duration of cancer (continuous variables) (Shayan, Shahkolahi, Ahmadlo, Vafaee, & Shayan, 2014). Groups of different sizes (n = 50, 100, 150, 200, 220) were randomly selected from the original population (n = 243). To increase the precision, resampling was repeated. Two data sets were ultimately produced and their classification indices were calculated. Data analysis was done using LDA and LR models from SPSS software (Statistical Package for the Social Sciences, Chicago, Illinois), version 16. LR was first used to determine the variables that affect depression and then the selection of best model was made based upon these variables.

### 3.1 Statistical Methods

#### 3.1.1 Logistic Regression Model

The binary LR model is used when the response variable takes just two values. This model is primarily used to identify the relationship between one or more independent variables (*X_i_*) and the dependent variable (*Y*) or to predict the independent variables that are most influential on the dependent variable.

The form of the LR determines the relationship between response probability and the predictor variables as:


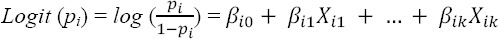


Or


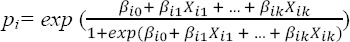


Where, 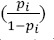 is the ratio of the probability of a success to the probability of a failure, called odds, *β_0_*, *β_i_* are parameters to be estimated, and *p*_i_ is the response probability for *i*th group, *k* is number of variables (Alkarkhi et al., 2008).

#### 3.1.2 Linear Discriminant Analysis

LDA predicts a categorical dependent variable using continuous or binary independent variables. Discriminant functions are linear combinations of variables useful when determining whether a set of variables is effective for predicting category membership. It is assumed that the variables have multivariate normal distribution and the variance/covariance matrices of the variables are homogeneous across groups. The form of the discriminant function is:


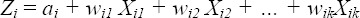


In this formula, the subscript *i* denotes the specific group; the subscripts 1, 2, …, *k* denote the *k* variables; *a_i_* is a constant, *w_ij_*, *j*=1, …, *k* is the weight for the *j*th variable in the computation of the classification score for the *i*th group; *X_j_* is the observed value for *j*th variable. *Z_i_* is the classification score. This formulation computes the classification scores for each case. The cases are then classified into specific groups based on the highest classification score.

#### 3.1.3 Comparison Criteria

The present study measured the B and Q indices and CE for accuracy of the prediction. The B and Q indices can be used to assess the accuracy of the outcome prediction.

The B index measures the average of squared difference between an estimated and actual value:


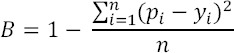


where p_i_ is a probability of classification into group i, y_i_ is the actual group membership (1 or 0), and n is the sample size of both groups. The B index lies between 0 and 1, where 1 denotes a perfect prediction. For random prediction in two equally-sized groups, the B index is 0.75.

The Q index is similar to the B index and is also a measure of predictive accuracy:


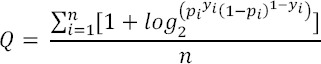


Q = 1 denotes a perfect prediction and a Q = 0 denotes a random prediction. When Q < 0 predictions are worse than random. If a predicted probability for the Q index equals 0 or 1, the Q index remains undefined (Pohar et al., 2004).

CE measures the percentage of incorrectly classified objects (misclassification). This index is determined from the results of LR and LDA. CE calculated one minus overall accuracy. The accuracy increases as the CE value decreases.

## 3. Results

The results of LR in the present study showed that satisfaction from her/his condition, a family history of depression, and duration of cancer were good predictors of depression in cancer patients. Comparisons between models were based on these variables. The results from two the series of samples are given in Tables 1 and 2. In these tables, it was assumed that the duration of cancer is continuous variable.

**Table 1:** Comparison of logistic and linear discriminant analysis based on classification indices with different sample sizes (the first random sampling)

n	Q		B		% Classification Error	
		
	LR	LDA	LR	LDA	LR	LDA
50	0.13907	0.13419	0.79099	0.79064	32	32
100	0.24627	0.24133	0.82432	0.82327	25	25
150	0.18272	0.16065	0.8024	0.79913	32.7	30.7
200	0.17096	0.15044	0.80163	0.79786	31	32.5
220	0.18768	0.16764	0.8056	0.80086	32.7	32.3
Total sample	0.17325	0.16053	0.80157	0.7988	31.7	32.9

**Table 2:** Comparison of logistic and linear discriminant analysis based on classification indices with different sample size (the second random sampling)

n	Q		B		% Classification Error	
		
	LR	LDA	LR	LDA	LR	LDA
50	0.09043	0.08933	0.78025	0.77949	36	34
100	0.20682	0.19196	0.81148	0.80863	27	28
150	0.19205	0.18664	0.80634	0.80602	30	30
200	0.17271	0.16254	0.80082	0.79909	32.5	34
220	0.17195	0.16205	0.80098	0.79913	31.4	32.7
Total sample	0.17325	0.16053	0.80157	0.7988	31.7	32.9

Table 1 summarizes the results of the first random sampling. The CE percentage, B and Q indices were calculated for different sample sizes with LR and LDA models. The results showed that the percentage of CE for LR is sometimes lower and sometimes higher than LDA.

Table 2 summarizes the results of the second random sampling. As seen, the CE percentage for LR is lower than LDA as the sample size increases. The results for the B and Q indices are very interesting; the LR model is more accurate than the LDA model. At a sample size of n = 50, the accuracy of prediction was low, especially for the LDA model, and the Q index is close to zero.

The duration variable was categorized and the calculations repeated. Table 3 provides the results based on the categorized variables and shows that the LR and LDA models are similar when based on CE. The results show that, as the sample size increased, the differences between the two methods for the B and Q indices became negligible.

**Table 3:** Effect of categorization of variables on classification indices with different sample sizes (based on the first random sampling)

n	Q		B		% Classification Error	
		
	LR	LDA	LR	LDA	LR	LDA
50	0.14323	0.14228	0.79442	0.79424	34	34
100	0.22965	0.22651	0.81936	0.81871	25	25
150	0.14863	0.14278	0.7939	0.79359	31.3	31.3
200	0.16303	0.16015	0.79902	0.79866	32	32
220	0.17487	0.16748	0.80141	0.8005	31.8	31.8
Total sample	0.16162	0.15824	0.79809	0.79781	32.5	32.5

## 4. Discussion

The present study investigated the effect of sample size and categorical variables on the accuracy of classification using different classification criteria. When both categorical and continuous variables were employed, the choice between models based on CE might be questionable. It appeared that LDA was more advantageous than LR in some cases and vice versa. These findings are in agreement with other studies. Baron (1991) concluded that LR performed better than the LDA when the data was non-normal, whereas little difference was found between models with multiple non-normal data sets in meta-analysis (Meshbane & Morris, 1996). Antonogeorgos et al. (2009) used LDA and LR to predict the presence of asthma symptoms. Their results showed that the correct classification rate differed slightly between LR and LDA, but that area under curve (AUC) was similar for both models. Marcos et al. (2010) presented an automatic obstructive sleep apnea syndrome detection algorithm based on classification of nocturnal oxygen saturation using LR and LDA. They showed that the overall accuracy and AUC were similar. Delmar et al. (2011) assumed multivariate normality and equal covariance matrices to estimate coefficients using LDA and LR that were identical. LDA and LR had the same true AUC, but the results of real data suggest that the finding is sensitive to the assumption of normality.

The results of the present study indicates that, when all variables were categorical, the LR and LDA models yielded similar results based on CE, but the B and Q indices produced more accurate results using LR. Pohar et al. concluded that when variables were normally distributed and categorized into a specific number of categories, the LDA model performed better if the number of categories was large enough (2004). CE was not an appropriate index because it was not sensitive to the accuracy of the prediction. The B and Q indices indicated that the LR model can provide more accurate predictions than the LDA in all situations. Although the use of CE is common, but it was not appropriate for the data used in this study and could cause bias.

The major drawback of LR is its requirement of a large sample size. Harrell and Lee (1985) showed that LDA was more accurate than LR for small samples. Another study of using real data found that LR worked better than DA for small samples (Johnston & Seshia, 1992). One study showed that sample size had a little effect on classification accuracy, although a small sample size had a greater effect on LR than on LDA (Fan & Wang, 1999). Pohar et al. (2004) showed that sample size had an evident effect on the difference between models.

In summary, the LR model is appropriate for prediction of depression in cancer patients based on the variable of satisfaction from her/his condition, family history of depression, and duration of cancer using B and Q indices. No effect was observed for sample size on classification for selection of the best model. When the sample size was small (n = 50), the accuracy of prediction was low especially for the Q index. It was shown that LDA can be used with small sample sizes.

## 5. Conclusion

The results of this study showed that logistic regression provided better results in some case and linear discriminant analysis in others, confirming that classification error is not appropriate. Although the B and Q indices provided better and more efficient criteria for discrimination between groups and better prediction when the purpose is to predict the response, the best choice is the model with higher accuracy. Further study should focus on classification error as it relates to mixture categorical and continuous variables.
